# Overview of Retention Strategies for Medical Doctors in Low- and Middle-Income Countries and Their Effectiveness: Protocol for a Scoping Review

**DOI:** 10.2196/52938

**Published:** 2024-01-08

**Authors:** Norehan Jinah, Ili Abdullah Sharin, Pangie Bakit, Izzuan Khirman Adnan, Kun Yun Lee

**Affiliations:** 1 Centre of Leadership & Professional Development Institute for Health Management National Institutes of Health Malaysia Shah Alam Malaysia

**Keywords:** health care workforce, retention strategies, medical doctors, low-income and middle-income countries, scoping review

## Abstract

**Background:**

The global shortage and maldistribution of health care workers, especially medical doctors, pose a significant threat to achieving the United Nations’ sustainable development goal 3 of ensuring well-being and healthy lives for all. Low- and middle-income countries (LMICs) are disproportionately affected by this crisis, with a high rate of brain drain from rural to urban areas, as well as to high-income countries. Various retention strategies have been implemented in different settings and organizations. However, their effectiveness remains underexplored, particularly in LMICs.

**Objective:**

We aim to review the available retention strategies for medical doctors in LMICs and to determine the effectiveness of the various strategies. This review aims to compile relevant research findings on this issue to generate a thorough summary of all the retention strategies practiced in LMICs and, more importantly, to provide the current state of evidence of the effectiveness of these strategies in retaining medical doctors in countries with limited resources and high disease burden.

**Methods:**

The structured framework given by Arksey and O'Malley will serve as the basis for conducting this scoping review. A comprehensive search strategy will be conducted across 4 electronic databases (PubMed, EBSCOHost, Scopus, and ScienceDirect). A systematic approach following the PRISMA-ScR (Preferred Reporting Items for Systematic Reviews and Meta-Analyses extension for Scoping Reviews) guidelines will be executed to search, screen, review, and extract data from studies that meet predefined inclusion criteria. Data encompassing bibliographical information, study location, retention strategies, influencing factors, and outcomes (effectiveness) will be obtained from the selected studies using standardized data extraction. Endnote and Microsoft Excel will be used for reference management and removal of duplicate studies. A narrative synthesis will be performed after categorizing and analyzing all the extracted data to identify recurrent themes.

**Results:**

This ongoing review will generate a comprehensive compilation of retention strategies implemented in LMICs to prevent brain drain among medical doctors. Data extraction is currently in progress, and completion is expected by early 2024. Themes regarding the types of strategies, influencing factors, and outcomes will be synthesized. The findings will highlight effective retention strategies, gaps, and challenges in implementation for the benefits of future research. By identifying common barriers and facilitators, this review will provide insights into enhancing the policies and initiatives for doctor retention in LMICs.

**Conclusions:**

This scoping review explores the retention strategies practiced in LMICs and attempts to identify effective strategies from existing research. By evaluating the barriers and challenges that influence the effectiveness of these strategies, policymakers and health care leaders can strive to obtain balanced and optimal health human resources in their respective organizations and countries.

**Trial Registration:**

Malaysian National Medical Research Register (NMRR) ID-23-01994-OGW; https://nmrr.gov.my/research-directory/ac4f5b88-8619-4b2b-b6c7-9abcef65fdcd

**International Registered Report Identifier (IRRID):**

DERR1-10.2196/52938

## Introduction

### Overview

Optimal functioning of the health care system relies heavily on the quantity and quality of health care workers (HCWs). The ability to enhance health service coverage and ensure that everyone in the population has access to the highest possible level of health relies on the availability, accessibility, acceptability, and quality of the health care workforce [1]. However, in recent years, a critical global challenge has emerged: a crisis characterized by a shortage and maldistribution of HCWs including doctors, nurses, and other professionals. This crisis poses a significant threat to achieving the United Nations Sustainable Development Goal 3 (promoting well-being and ensuring healthy lives for people of all ages) [2]. Nowhere is the impact of this challenge more acute than in low- and middle-income countries (LMICs), where limited financial and human resources compound the challenge of providing essential health care services. LMICs represent various nations in a diverse economic spectrum, encompassing low-income, lower-middle-income, and upper-middle-income countries. On the basis of the World Bank classification [3], low-income economies are defined as those with a gross national income (GNI) per capita of ≤US $1135 in 2022; lower middle-income economies are those with a GNI per capita between US $1136 and US $4465; upper middle-income economies are those with a GNI per capita between US $4466 and US $13,845. In contrast, high-income economies are those with a GNI per capita of ≥US $13,846. Remarkably, this classification encompasses 137 countries, constituting 63% of all nations worldwide [4].

The World Health Organization projected that by 2030, countries, predominantly LMICs, will face a substantial deficit of approximately 18 million health workers [5]. This significant shortage will greatly hamper the capacity to deliver vital health care services to those populations in most significant need. Simultaneously, countries with varied levels of socioeconomic development encounter different challenges in health human resource planning related to employment, deployment, and retention of their workforce [6]. Medical doctors are vital to the health care system because of their expertise, care, and impact. They play a crucial role in ensuring optimal health care delivery within health care institutions [7]. However, many parts of the world are grappling with a shortage of doctors, which stems from various factors such as emigration, imbalanced distribution between rural and urban areas, and shifts in population demographics [5]. There is a global shortage of approximately 2.8 million doctors [5], with LMICs bearing the brunt of this burden [8]. This scarcity is further exacerbated by the phenomenon of brain drain, with doctors from LMICs emigrating to high-income countries (HICs) due to better job offers and career progress. In some HICs, foreign-trained physicians sometimes amount to one-fifth of the total number of doctors in the workforce [9]. The movement of doctors from lower-to higher-income settings has resulted in substantial economic consequences, not solely due to the transfer of human capital, but more importantly, indirect impacts, such as increased morbidity and mortality associated with the loss of doctors [10].

Apart from brain drain to other countries, there is also a high rate of doctors’ resignations from the public health care system to join the more lucrative private sector, especially in countries with dual health care financing systems. Job dissatisfaction, including unsatisfactory work environment (lack of facilities, inflexible working hours, poor career progression, lack of professional autonomy, and ineffective management style) and unfavorable service conditions (poor salaries and funding, duplication of activities), are closely associated with high mobility, especially from the public to private sectors [11-13]. The phenomenon of HCWs resigning poses a significant obstacle to the advancement of the health care system in any given country, making it a topic of widespread concern [14]. The increasing number of resignations among HCWs, particularly in the Asia Pacific region, has been reported as the greatest threat to the development and sustainability of a resilient health care system in a recent study [15]. Despite efforts to increase supply and retain them, the workforce is still struggling to meet public health demands, as demonstrated in Spain and Brazil [16]. The same issue was also reported in India, where the vacancy rates were nearly 21% and 42% for medical officers and specialists at health centers, respectively [17].

Addressing the global health workforce crisis requires comprehensive strategies at both national and international levels. Retaining HCWs is a challenge in almost every country, be it HICs such as Canada, Australia, and Scotland or LMICs in Africa and Asia, especially in rural and remote areas [18,19]. Retention encompasses the duration between the initial engagement with a service and the eventual separation or departure from that service. It serves as a metric to gauge the length of time an individual stays within the service [20]. Retention strategies in the context of doctors encompass a range of interventions designed to attract and keep doctors in particular settings, such as remote or rural areas, with a specific focus on LMICs [21-23]. These strategies are aimed at mitigating doctor shortages and ensuring equitable health care access for underserved populations. Policy makers and health care managers must comprehend the factors that influence doctor retention and formulate targeted measures to address these factors [24,25]. Effective retention strategies contribute to the stability and continuity of health care delivery, especially in regions with limited accessibility [21,23].

The significance of retention strategies lies in their capacity to yield various benefits, including cost savings, employee engagement, productivity, knowledge retention, competitive advantage, and organizational stability [26]. Addressing doctor shortages requires tailoring retention strategies to the unique challenges and requirements of health care professionals in each country. This is particularly critical in LMICs, where health care systems often contend with fragility, staffing shortages, limited resources, and a higher disease burden [27-29]. Furthermore, these countries grapple with brain drain challenges, issues of health care accessibility, weakened political will, and unstable governmental systems [30-33].

There are many known impediments to the retention of doctors, the most common being unfavorable working conditions, limited opportunities for career advancement, nonappealing financial incentive structures, unsupportive community environments, and the restriction of financial resources [34,35]. Other barriers include inadequate living standards, excessive workloads, insufficient equipment, lack of opportunities for skill enhancement and private practice, and unfair promotion practices [36]. In addition, stress, burnout, and insufficient work-life balance also play a role in doctors’ decision to leave [37]. Strategies aimed at addressing these barriers have been proposed and implemented at various levels and organizations, such as providing career development plans, ensuring minimum financial incentives, establishing avenues for private practice, enhancing work conditions, providing opportunities for skill improvement, and implementing transparent and equitable promotion systems.

### Objective of Conducting the Scoping Review

Numerous publications have discussed the factors influencing the retention of doctors in LMICs [38-41], providing suggestions for various strategies and initiatives. However, there is limited research evaluating and summarizing the effectiveness of these strategies, particularly in LMICs. Therefore, the objective of this scoping review is to identify and delineate the available retention strategies for medical doctors in LMICs and to determine the effectiveness of these strategies.

To determine if prior research has addressed the same subject, we performed an initial exploratory literature review. Our search revealed the absence of existing or ongoing systematic reviews and scoping reviews related to our specific topic. McClain et al [42] primarily explored retention strategies and barriers concerning nurses, while Noya et al [43] concentrated on the rural and remote medical workforce, and Verma et al [22] focused on primary care doctors in general.

Conversely, our review aims to synthesize research evidence to generate an all-encompassing perspective on the effectiveness of retention strategies for doctors in LMICs. This synthesis will identify gaps in existing literature, pinpointing areas that require additional investigation within the context of doctor retention in resource-constrained countries with high disease burden. Our inclusive methodology considers a broad spectrum of studies and settings and delivers a comprehensive evaluation of these strategies.

## Methods

### Ethical Considerations

As the methodology for this scoping review solely entails reviewing and collecting data from existing literature without involving human participants, ethical clearance was waived by the Medical Research and Review Committee Malaysia.

### Protocol Design

#### Overview

For this scoping review, we will use the methodological framework introduced by Arksey and O'Malley [44], who structured the review process into 5 stages. In addition, we enhanced the quality and rigor of our review based on the guidelines from the Joanna Briggs Institute Manual [45]. We will also incorporate the recommendations provided by Levac et al [46] to ensure consistency in assessing the studies during this scoping review. Transparent reporting will be ensured by using the PRISMA-ScR (Preferred Reporting Items for Systematic Reviews and Meta-Analyses extension for Scoping Reviews) guidelines [47]. We describe the protocol for this scoping review in five stages:

1. Formulating research questions

2. Identifying relevant studies

3. Selecting eligible studies

4. Charting the data

5. Collating, summarizing, and reporting the results

#### Stage 1: Formulating Research Questions

Following the recommendations given by Levac et al [46], we set our objective to explore strategies or interventions available for retaining doctors within health care institutions in LMICs and to identify effective measures to prevent doctor attrition. Therefore, we formulated two specific research questions for this review:

1. What are the retention strategies currently being implemented for doctors in LMICs?
2. Which strategies have been identified and evaluated as effective in retaining doctors in LMICs?

#### Stage 2: Identifying Relevant Studies

A meticulous search strategy plays a vital role in ensuring the inclusion of pertinent studies in scoping reviews. The research team has developed a comprehensive search strategy that encompasses various keywords and their synonyms related to the topic of interest. We selected search terms based on the research questions, including terms such as “retention,” “retain,” “maintain,” “doctor,” “physician,” and “general practitioner.” These terms has been used both individually and in combination following the iterative process inherent in the scoping review methodology.

The final search string, adhering to Boolean logic, takes the following form: *(retention OR retain OR intention to leave OR intention to stay OR motivation to stay OR willingness to work) AND (doctor OR physician OR specialist OR general practitioner OR medical practitioner) AND (low- and middle-income countries OR LMIC)*. This meticulously designed search string aimed to gather all pertinent materials aligned with the objectives of this scoping review.

Various types of documents were screened during this stage, including journal articles, documents, or regulatory reviews, sourced from each of the 4 databases: PubMed, EBSCOHost, Scopus, and ScienceDirect. These databases were selected for their relevance to health and human resource services. During the screening process, if the available information in the title and abstract is insufficient to make an informed decision, the articles will be included for full-text screening. Adhering to the standard approach for conducting scoping reviews, we will not conduct quality appraisal of the included studies. An example of a preliminary MEDLINE (PubMed) search strategy is presented in Textbox 1.

**Example of MEDLINE (PubMed) search strategy.**

- (retention[Title/Abstract] OR retain[Title/Abstract] OR intention to stay[Title/Abstract] OR intention to leave[Title/Abstract] OR motivation to stay[Title/Abstract] OR willingness to work[Title/Abstract]) AND (doctor*[Title/Abstract] OR physician*[Title/Abstract] OR specialist*[Title/Abstract] OR general practitioner*[Title/Abstract] OR general physician*[Title/Abstract] OR medical practitioner*[Title/Abstract]) AND (low- and middle-income country[Title/Abstract] OR low- and middle-income countries[Title/Abstract] OR low-income country[Title/Abstract] OR low-income countries[Title/Abstract] OR lower middle-income country[Title/Abstract] OR lower middle-income countries[Title/Abstract] OR upper middle-income country[Title/Abstract] OR upper middle-income countries[Title/Abstract] OR Afghanistan[Title/Abstract] OR Albania[Title/Abstract] OR Algeria[Title/Abstract] OR American Samoa[Title/Abstract] OR Angola[Title/Abstract] OR Armenia[Title/Abstract] OR Azerbaijan[Title/Abstract] OR Bangladesh[Title/Abstract] OR Belarus[Title/Abstract] OR Byelarus[Title/Abstract] OR Belorussia[Title/Abstract] OR Belize[Title/Abstract] OR Benin[Title/Abstract] OR Bhutan[Title/Abstract] OR Bolivia[Title/Abstract] OR Bosnia[Title/Abstract] OR Botswana[Title/Abstract] OR Brazil[Title/Abstract] OR Bulgaria[Title/Abstract] OR Burma[Title/Abstract] OR Burkina Faso[Title/Abstract] OR Burundi[Title/Abstract] OR Cabo[Title/Abstract] Verde[Title/Abstract] OR Cape Verde[Title/Abstract] OR Cambodia[Title/Abstract] OR Cameroon[Title/Abstract] OR Central African Republic[Title/Abstract] OR Chad[Title/Abstract] OR China[Title/Abstract] OR Colombia[Title/Abstract] OR Comoros[Title/Abstract] OR Comoros[Title/Abstract] OR Comoro[Title/Abstract] OR Congo[Title/Abstract] OR Costa Rica[Title/Abstract] OR Côte d'Ivoire[Title/Abstract] OR Cuba[Title/Abstract] OR Djibouti[Title/Abstract] OR Dominica[Title/Abstract] OR Dominican Republic[Title/Abstract] OR Ecuador[Title/Abstract] OR Egypt[Title/Abstract] OR El Salvador[Title/Abstract] OR Equatorial Guinea[Title/Abstract] OR Eritrea [Title/Abstract]OR Ethiopia[Title/Abstract] OR Fiji[Title/Abstract] OR Gabon[Title/Abstract] OR Gambia[Title/Abstract] OR Gaza[Title/Abstract] OR Georgia[Title/Abstract] OR Georgia Republic[Title/Abstract] OR Ghana[Title/Abstract] OR Grenada[Title/Abstract] OR Grenadines[Title/Abstract] OR Guatemala[Title/Abstract] OR Guinea[Title/Abstract] OR Guinea[Title/Abstract]- Bissau[Title/Abstract] OR Guyana[Title/Abstract] OR Haiti[Title/Abstract] OR Herzegovina[Title/Abstract] OR Hercegovina[Title/Abstract] OR Honduras[Title/Abstract] OR India[Title/Abstract] OR Indonesia[Title/Abstract] OR Iran[Title/Abstract] OR Iraq[Title/Abstract] OR Ivory Coast[Title/Abstract] OR Jamaica[Title/Abstract] OR Jordan[Title/Abstract] OR Kazakhstan[Title/Abstract] OR Kenya[Title/Abstract] OR Kiribati[Title/Abstract] OR Democratic People’s Republic of Korea[Title/Abstract] OR Kosovo[Title/Abstract] OR Kyrgyz[Title/Abstract] OR Kirghizia[Title/Abstract] OR Kirghiz[Title/Abstract] OR Kyrgyzstan[Title/Abstract] OR Lao PDR[Title/Abstract] OR Laos[Title/Abstract] OR Lebanon[Title/Abstract] OR Lesotho[Title/Abstract] OR Liberia[Title/Abstract] OR Libya[Title/Abstract] OR Macedonia[Title/Abstract] OR Madagascar[Title/Abstract] OR Malawi[Title/Abstract] OR Malay[Title/Abstract] OR Malaya[Title/Abstract] OR Malaysia[Title/Abstract] OR Maldives[Title/Abstract] OR Mali[Title/Abstract] OR Marshall Islands[Title/Abstract] OR Mauritania[Title/Abstract] OR Mauritius[Title/Abstract] OR Mexico[Title/Abstract] OR Micronesia[Title/Abstract] OR Moldova[Title/Abstract] OR Mongolia[Title/Abstract] OR Montenegro[Title/Abstract] OR Morocco[Title/Abstract] OR Mozambique[Title/Abstract] OR Myanmar[Title/Abstract] OR Namibia[Title/Abstract] OR Nepal[Title/Abstract] OR Nicaragua[Title/Abstract] OR Niger[Title/Abstract] OR Nigeria[Title/Abstract] OR Pakistan[Title/Abstract] OR Palau[Title/Abstract] OR Papua New Guinea[Title/Abstract] OR Paraguay[Title/Abstract] OR Peru[Title/Abstract] OR Philippines[Title/Abstract] OR Principe[Title/Abstract] OR Romania[Title/Abstract] OR Ruanda[Title/Abstract] OR Rwanda[Title/Abstract] OR Samoa[Title/Abstract] OR Sao Tome[Title/Abstract] OR Senegal[Title/Abstract] OR Serbia[Title/Abstract] OR Sierra Leone[Title/Abstract] OR Solomon Islands[Title/Abstract] OR Somalia[Title/Abstract] OR South Africa[Title/Abstract] OR South Sudan[Title/Abstract] OR Sri Lanka[Title/Abstract] OR St Lucia[Title/Abstract] OR St Vincent[Title/Abstract] OR Sudan[Title/Abstract] OR Surinam[Title/Abstract] OR Suriname[Title/Abstract] OR Swaziland[Title/Abstract] OR Syria[Title/Abstract] OR Syrian Arab Republic[Title/Abstract] OR Tajikistan[Title/Abstract] OR Tadzhikistan[Title/Abstract] OR Tajikistan[Title/Abstract] OR Tadzhik[Title/Abstract] OR Tanzania[Title/Abstract] OR Thailand[Title/Abstract] OR Timor[Title/Abstract] OR Togo[Title/Abstract] OR Tonga[Title/Abstract] OR Tunisia[Title/Abstract] OR Turkey[Title/Abstract] OR Turkmen[Title/Abstract] OR Turkmenistan[Title/Abstract] OR Tuvalu[Title/Abstract] OR Uganda[Title/Abstract] OR Ukraine[Title/Abstract] OR Uzbek[Title/Abstract] OR Uzbekistan[Title/Abstract] OR Vanuatu[Title/Abstract] OR Venezuela[Title/Abstract] OR Vietnam[Title/Abstract] OR West Bank OR Yemen[Title/Abstract] OR Zambia[Title/Abstract] OR Zimbabwe[Title/Abstract])

#### Stage 3: Selecting Eligible Studies

The review process begins with the team convening to discuss decisions related to study inclusion and exclusion based on the principles of transparency, reproducibility, and rigor. This practice further advances a systematic and unbiased approach throughout the review process. The inclusion and exclusion criteria are presented in Textbox 2. We chose to focus primarily on studies published in English language due to their global prevalence, ensuring a comprehensive analysis, increased accessibility, and reduced language-related biases due to limited translation resources. Furthermore, the focus on studies published in English language streamlines the accessibility and application of research findings, making them readily available and applicable to a broader audience.

To maintain the scientific rigor of this review, we made a deliberate choice to exclude gray literature from our review, such as dissertations, essays, consensus, reports, theses, and government documents. However, while gray literature may provide valuable insights that supplement traditional academic literature [48], it presents challenges in terms of systematic search and quality verification [49].

Following the PRISMA-ScR guidelines [47], the first step begins with identifying articles from various databases. Duplicates and irrelevant studies will then be removed. Abstracts or full texts will be evaluated based on predetermined inclusion and exclusion criteria to determine eligible studies. This screening process involves careful examination of both the retrieved search results and their reference lists. To ensure the most relevant search results, we will refine the literature search throughout the review process. At least 2 investigators will independently assess the eligibility of publications by reviewing their titles and abstracts. Publications deemed relevant to this scoping review are obtained in full text and reviewed against the same inclusion criteria.

**Figure 1:**
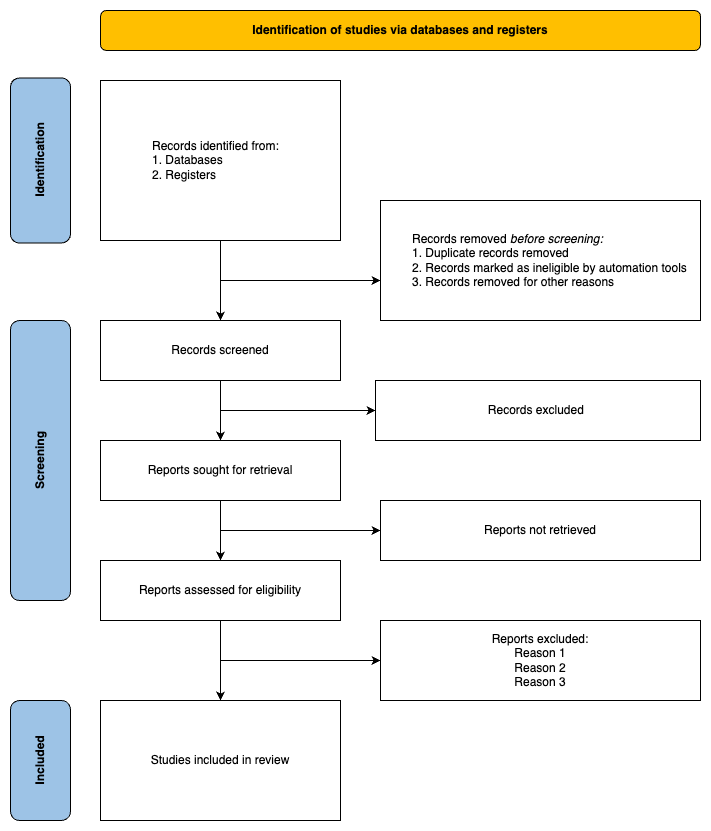
PRISMA (Preferred Reporting Items for Systematic Reviews and Meta-Analyses) flow diagram for the scoping review process.

In cases of disagreement during publication selection, both reviewers will revisit the full-text articles to reach a consensus. If consensus cannot be reached, an impartial third reviewer will be consulted to resolve the disagreement. Consistent meetings and discussions at different stages of the article review process are essential to maintain alignment, address challenges, refine search strategies, ensure consistency, and foster a collaborative and efficient approach. The scoping review will record and report reasons for excluding sources of evidence in the full text that do not meet the inclusion criteria. The reporting of the review will incorporate a PRISMA (Preferred Reporting Items for Systematic Reviews and Meta-Analyses) flow diagram (Figure 1), which visually presents the screening and selection process [50].

**Inclusion and exclusion criteria of the study selection process.**

**Inclusion criteria**

- Publication year: January 2013 to February 2023
- Language in publication: English
- Research location: low- and middle-income countries (LMICs)
- Target population: medical doctors
- Types of documents: journal articles, documents, or regulatory reviews with proper references

**Exclusion criteria**

- Publication year: before January 2013 and after February 2023
- Language in publication: other languages
- Research location: other than LMICs
- Target population: other health care professionals
- Types of documents: dissertations, essays, consensus, government documents, reports, and theses that do not have any proper references

#### Stage 4: Charting the Data

The data extracted from the full-text articles will be organized into a data extraction table using Microsoft Excel (Microsoft Corporation). The data table will be structured to accommodate the characteristics of the data. The aim of charting the data is to create a descriptive summary of the results to address the objectives of the scoping review and to answer the research questions. This process facilitates the categorization of information before proceeding with further tabulation. For reference, Textbox 3 presents the categories corresponding to each characteristic in the data extraction table. In an iterative process, investigators will continually gather data and keep the data extraction table up-to-date. If significant data are found in records initially not designated for extraction, the data extraction form will be revised, and these additional data will be retrieved from the records already reviewed.

**Preliminary data extraction table.**

**Basic characteristics and description**

- Bibliographical data
  First author and year of publication of the article
- Article title
  A succinct description of the content of the article
- Country
  Name of the low- and middle-income countries
- Aims or purpose of the study
  Expresses the intention or aspiration of the research
- Type of study
  Study design or methodology
  Which type of study was conducted?
- Study population
  Physician—specialty or department
  Number of people involved
  Inclusion and exclusion criteria of the study
  Demographic characteristics
  Other characteristics
- Study location
  Location characteristics (urban, rural, or remote or hospital or district, state, or area)
  Institution (name)
- Factors influencing retention
  Financial or career and professional or working conditions, personal, cultural, or living conditions factors
- Retention strategy
  Strategy type or focus (education and regulatory or ii. monetary compensation or iii. management, and environment and social support)
  Strategy name
  Strategy characteristics, content, and description
  Strategy implementation (levels, duration, and date)
- Outcomes measure
  Description of the result (effective or successful to retain)
  How were the turnover and results assessed?
- Barriers and challenges
  Barriers and challenges in implementing the strategies
- Study limitations
  Weaknesses within the research design that may influence the outcomes and conclusions of the research

#### Stage 5: Collating, Summarizing, and Reporting the Results

The primary goal of the scoping review is to present the narrative findings of existing literature through an analytical framework or thematic construction, without the requirement to assess the quality or significance of each study. We will use a traditional integrative review approach to compile all the identified materials. Our objective is to identify recurring themes across research and synthesize data from the selected studies. Using these themes as guidelines, we will create a literature map and present it in the form of a table, summarizing the publications and their respective characteristics.

The results of the scoping review will be organized into tables that categorize the characteristics of each publication. Accompanying these results will be narrative summaries that describe how each result relates to our research questions, including any unexpected or particularly notable findings. We will also address any gaps observed in the literature, research needs, and implications for practice. Subsequently, the outcomes of this review will be shared with relevant stakeholders, and their expertise and perspectives will be incorporated.

## Results

This review will provide a comprehensive mapping of existing research and literature pertaining to the retention of medical doctors in LMICs to enhance the understanding of the complex dynamics of doctor retention. It will also assess the current knowledge and pinpoint any gaps in the literature, focusing on factors influencing doctor retention and effective retention strategies such as financial incentives, working conditions, career advancement opportunities, and personal motivations.

Furthermore, this review can offer insights into best practices and approaches for retaining doctors in LMICs to guide policy makers and health care administrators who struggle with retention challenges. They can customize the best policy recommendations based on specific needs and obstacles in local settings to improve doctor retention rates in their respective organizations and governments.

The review was initiated in May 2023, and the research protocol was finalized in July 2023. We registered the review with the Malaysian National Medical Research Register (NMRR ID-23-01994-OGW). The search, which was concluded in August 2023, yielded 9141 articles. The PRISMA flow diagram will be used to illustrate the flow of the literature search in this review [50]. The results will be presented using charts and tables, supplemented by a narrative description. Any existing literature gaps will be identified, and the significance of our findings will be emphasized in the subsequent discussion section. The review is expected to be concluded in January 2024, with the outcomes published in a journal for wider dissemination.

## Discussion

### Overview

Adequate investment in health care capacity is imperative to move toward the United Nations’ sustainable development goals, specifically goal 3 (ensuring good health and well-being) and goal 10 (reducing inequalities), and to achieve various global development objectives, with a robust health care workforce being the top priority. Therefore, establishing a comprehensive plan that encompasses effective retention strategies to complement medical education reforms is vital to cultivating a health care environment that is equitable and resilient at both regional and global levels. Our focus on retention strategies for medical doctors is driven by their unique challenges and critical roles in health care. Doctors hold central positions in health care delivery, not only providing medical expertise but also taking a leadership role in influencing critical patient care decision-making, and their turnover can have significant negative impacts on patient care and quality of health care services [51]. Furthermore, doctors are the most affected by the brain drain crisis, especially in LMICs, leading to a significant financial burden and experience loss. Therefore, prioritizing doctor retention is vital for mitigating brain drain, reducing productivity and financial loss, and sustaining effective health care service delivery.

The shortage of doctors in LMICs represents a pressing concern that demands immediate attention and concerted efforts on a global scale, in view of its significant impact on public health. This predicament has a direct adverse effect on the health and welfare of populations residing in LMICs, as doctor shortages can impede access to crucial medical services, ultimately resulting in preventable illnesses and higher mortality rates. Moreover, cross-border brain drain exacerbates existing health care inequalities both within and between countries. Persistent disparities in the accessibility of health care services, if they continue to exist, will disproportionately affect rural and underserved areas with limited resources, thereby perpetuating social and economic inequalities and impeding advancements toward achieving universal health care coverage.

### Expected Outcomes

This scoping review will present a comprehensive overview of retention strategies that have been proposed, practiced, and evaluated in LMICs as a response to overcome the challenges faced in retaining medical doctors and preventing brain drain. These strategies may encompass a wide array of approaches, including financial incentives, opportunities for professional development, initiatives to promote work-life balance, and support for career advancement. Moreover, the focus on LMICs may shed light on distinct regional or country-specific challenges and variations in customized strategies. It may also highlight the varied effectiveness of different strategies, depending on the contextual factors at play. It is unlikely to be a one-size-fits-all solution, as certain strategies may exhibit promising outcomes in bolstering medical doctor retention, while others may demonstrate limited impact depending on the local settings.

In short, this review will present common barriers and facilitators that significantly influence the successful implementation of retention strategies for doctors in LMICs. By exploring the challenges encountered during strategy implementation, we also aim to offer a more comprehensive and nuanced understanding of the factors influencing the effectiveness of doctor retention strategies in LMICs. This, in turn, can contribute to improving the retention of medical doctors in LMICs, aligning with the Sustainable Development Goal 3 goal of promoting well-being and ensuring healthy lives for everyone. Comprehension of these elements has the potential to aid policy makers and health care administrators in developing more relevant interventions and prioritizing effective strategies.

Since it is likely that different contexts play a critical role in the outcomes of various retention strategies, we will also attempt to address this connection in our review. Certain strategies, if proven successful, can also be modified and embedded within a broader health care ecosystem to benefit a wider group of health care professionals. Common factors contributing to brain drain among HCWs include financial rewards, career development, hospital infrastructure, political issues, and family issues [52]. While we focus on the dynamics surrounding medical doctors and the customized retention approach for them in this review, as the challenges faced by doctors may be unique and differ significantly from those of other groups, the comparison and extrapolation of various retention strategies for different health care professionals is a worthy topic for future research or review.

### Review Limitations

This review has several limitations that deserve further discussion. First, the language restriction used in the search strategy may have unintentionally excluded relevant studies published in languages other than English. This is a significant concern because many LMICs have diverse linguistic landscapes with numerous languages. The decision to focus primarily on English was necessitated by practical considerations, such as the broader availability and accessibility of English-language research. Furthermore, we believe that the exclusion of non–English-language studies would minimize language-related biases in the review process, given the limited access to translation resources in our setting.

Another limitation of this review is the exclusion of gray literature. This decision is influenced by the difficulties associated with accessing gray literature, which encompasses issues of limited availability, inconsistent indexing, variable accessibility, and challenges in assessing the quality and reliability of information. By excluding gray literature, there is a risk of missing important findings and diverse perspectives not found in peer-reviewed academic sources. Nevertheless, although gray literature can offer valuable insights as a complement to conventional academic literature [48], it introduces difficulties in systematic retrieval and quality assessment [49], thus making it difficult to maintain the scientific rigor of this review.

In addition, this review is likely to include studies with different levels of methodological rigor and quality, and this could potentially affect the overall reliability of its conclusions and may introduce heterogeneity into our analysis. Moreover, this review aims to provide a comprehensive overview of existing literature regarding effective retention strategies for doctors in LMICs; thus, the analysis results are likely to be less in depth compared with systematic reviews that follow a more rigorous and narrowly focused methodology. Nonetheless, this broad approach is valuable for summarizing the diversity of strategies and findings in the field of doctor retention in LMICs, allowing for a holistic understanding of the subject.

### Conclusions

This scoping review is fundamental in providing a better understanding of the practical implications of various retention strategies for doctors in LMICs and in drawing valuable lessons from effective strategies in existing literature. Furthermore, by highlighting emerging trends and identifying implementation challenges within LMICs, this review will pave the way for more precisely targeted policies and interventions to strengthen doctor retention in the most needed regions. It also offers valuable guidance to policy makers and health care administrators by showcasing best practices with positive outcomes, thereby refining their approach to addressing attrition and brain drain.

## Abbreviations

GNI: gross national income
HCW: health care worker
HICs: high-income countries
LMICs: low- and middle-income countries
PRISMA: Preferred Reporting Items for Systematic Reviews and Meta-Analyses
PRISMA-ScR: Preferred Reporting Items for Systematic Reviews and Meta-Analyses extension for Scoping Reviews

## Data Availability

Data sharing is not applicable to this paper, as no data sets were generated or analyzed in this review.
